# Current basis and future directions of zebrafish nutrigenomics

**DOI:** 10.1186/s12263-019-0658-2

**Published:** 2019-12-27

**Authors:** Michael B. Williams, Stephen A. Watts

**Affiliations:** Department of Biology, University of Alabama at Birmingham, Birmingham, AL 35294 USA

**Keywords:** Zebrafish, Nutrition, Diet, Nutrigenomics, Nutrigenetics, Gene Interactions

## Abstract

This review investigates the current state of nutrigenomics in the zebrafish animal models. The zebrafish animal model has been used extensively in the study of disease onset and progression and associated molecular changes. In this review, we provide a synopsis of nutrigenomics using the zebrafish animal model. Obesity and dyslipidemia studies describe the genomics of dietary-induced obesity in relation to high-fat/high-calorie diets. Inflammation and cardiovascular studies describe dietary effects on the expression of acute inflammatory markers and resulting chronic inflammatory issues including atherosclerosis. We also evaluated the genomic response to bioactive dietary compounds associated with metabolic disorders. Carbohydrate metabolism and β-cell function studies describe the impacts of high-carbohydrate dietary challenges on nutritional programming. We also report tumorigenesis in relation to dietary carcinogen exposure studies that can result in permanent genomic changes. Vitamin and mineral deficiency studies demonstrate transgenerational genomic impacts of micronutrients in the diet and temporal expression changes. Circadian rhythm studies describe the relation between metabolism and natural temporal cycles of gene expression that impacts health. Bone formation studies describe the role of dietary composition that influences bone reabsorption regulation. Finally, this review provides future directions in the use of the zebrafish model for nutrigenomic and nutrigenetic research.

## Introduction

### Nutrigenomics and nutrigenetics

The intake of specific food items and specific nutrients, or their absence from the diet, has long been related to the manifestation of disease states in humans and animals [1]. As we have gained an understanding of the roles of more specific nutrients and nutrient levels needed to maintain normal life function and prevent deficiency- or toxicity-related diseases, we now understand important nutrient-gene interactions (nutrigenomics) and how variation in individual genetics affects nutrient requirements and tolerances, requiring personalized diet recommendations (nutrigenetics).

Nutrigenomics can be defined as two alternate mechanisms of nutrient-gene interactions [2]. First, dietary components can act to alter gene expression or act as cofactors within metabolic systems. The impacts of these interactions of dietary components and genes are temporary and rely on the presence of the dietary component with effects modulated by the availability and storage of these components. These effects are more readily observed in poorly stored nutrients such as water-soluble vitamins. Second, there are longer-lasting impacts on a gene by diet interactions such as dietary components that alter mutation rates and result in essentially permanent genome alterations, or components that alter genome methylation patterns resulting in heritable alterations of the genome [3–5]. The ability to make personalized dietary recommendations based on a person’s individual genome could be considered an “ultimate goal” in the clinical application of nutrigenomic research, similar to the goal of personalized medicine [6]. To better understand these complex networks of nutrient and gene interactions, researchers can use modern “omics” technologies as well as novel high-throughput translational models. One increasingly more commonly utilized model for this type of experimentation is the zebrafish.

### Danio rerio

The zebrafish species *Danio rerio* has become the third most utilized NIH animal model behind mice and rats [7]. There are important reasons for its utilization, both logistical and translational. Aside from a fast development time and easy and cost-efficient husbandry, the fully sequenced and annotated genome shows high similarity to humans, even accounting for a historical whole-genome duplication event in teleosts [8]. An abundance of molecular techniques has been developed for the model including CRISPR, GFP models, and Cre-Lox models [9–11]. Perhaps most important to nutrigenomics research is the similarity in how zebrafish metabolize and deposit dietary fat [12]. A high-fat diet was found to affect adipose gene expression (as measured by microarray) more similarly between humans and zebrafish than between humans and mice or rats [12]. This indicated a potential for better translational research into metabolic syndrome using this model, to be discussed later in detail below.

## Nutrition-related studies

### Obesity and dyslipidemia

Obesity has been a growing concern in the western world, and now worldwide. The age-standardized prevalence of obesity was 39.6% between 2015 and 2016 [13]. Obesity is often associated with several comorbidities including cardiovascular disease, diabetes, hypertension, and dyslipidemia [14]. Individuals with an obese phenotype display dysregulation of multiple genes [15]. These genes have been reported to be involved with metabolic processes and inflammatory responses.

Animals that over ingest calories (usually via a high-carb or high-fat diet) leading to stored adipose are considered models of dietary-induced obesity (DIO) and can be utilized to better understand physiological or molecular events that occur as a result of obese phenotypes [16]. The first seminal research for zebrafish DIO was reported by Oka et al. [12]. This study investigated DIO by over- or underfeeding *Artemia* (a live diet commonly used in zebrafish culture), or by caloric restriction following overfeeding. Overfeeding zebrafish (feeding ad libitum) resulted in an increase of fish BMI (measured as g weight/cm^2^ length by the investigators), plasma triglycerides, and hepatic steatosis in both males and females. Along with these metrics of growth and metabolic health, 168 genes were dysregulated. Following caloric restriction, fish weight and plasma triglycerides decreased significantly and 97 genes that had been dysregulated were normalized. Genes that were affected by overfeeding were in ontologies of blood coagulation, triglyceride metabolism, platelet activation, fatty acid metabolism, and cholesterol efflux. Comparisons of genes with expressions changed by DIO in zebrafish, rats, mice, and humans show a strong similarity in metabolic pathways involved among these species.

Landgraf et al. examined the effects of increased dietary calories leading to an obese phenotype by overfeeding either a high-fat diet (HFD-OF) or a normal fat diet (NFD-OF) [17]. This was evaluated only in adult male zebrafish and following 8 weeks both treatments gained weight and had increased percent body fat compared to a non-overfed control group (NFD). The HDF-OF, however, added on less body weight and no difference in body fat percentage compared to NFD-OF. HDF-OF showed differences in markers of metabolic health, namely increased fasting blood glucose, plasma triglycerides, and cholesterol compared to NFD-OF or NFD. Following from changes in blood glucose, there was an increased ratio of Thr 307 (human Thr 308 ortholog site) pAKT/AKT by western blot in the liver suggesting early insulin resistance [18]. Expression of *pparg* and *lpl*, lipid metabolism genes in the adipose, and *srebf1*, which regulates cholesterol biosynthesis in the liver, was reduced in the HFD-OF compared to the NFD-OF. Expression of *fabp11a*, lipid metabolism gene, and *col1a1a*, a collagen gene contributing to fibrosis [19] in the liver, was increased in HDF-OF compared to NFD-OF. This study demonstrates the effectiveness of the zebrafish model for investigating different metabolic and body composition phenotypes induced by different diets. Current evidence strongly supports *ppar* isoforms present having similarity in regulating mechanism for lipid metabolism as well as high structural similarity (67–74% identity) among humans, mice, and zebrafish [20]. High dietary linolenic acid increased expression of *fabp2* in the intestine and *fabp3* in the liver [21]. These genes are predicted to be regulated via *ppar* isomers.

As an alternative to evaluating zebrafish with a DIO phenotype, fasting experiments can be used to determine how energy and nutrients are allocated and absorbed. Fasting in humans has shown to have health benefits in obese individuals with either caloric restriction or intermittent fasting [22]. In addition to proven clinical benefits, studies of human fasting have provided a better understanding of genes that regulate energy intake and allocation. The same may be true for use of the zebrafish model. Zebrafish fasted for 3 weeks had reductions in weight, blood glucose, and liver glycogen, cholesterol, triglycerides, and phospholipids [23]. Fasted zebrafish also showed changes in the gene expression of *srebp1+2*, *mtor*, *ampk*, and *crebp3l3*, all known regulators of cellular energy and growth. Activity of proteins related to protein and sugar metabolism was also affected. Most effects were significantly ameliorated following a refeeding period, while some only trended towards the initial prefasting state. Similar zebrafish studies have evaluated dietary restriction (DR). DR, with either restricted calories, macronutrients, or feed-timing restriction, is one of the only known potent cross-species intervention that extends the life span [24, 25]. Arslan-Ergul et al. provided a control or calorie-restricted diet to either young (8–8.5 months) or old (26–32.5 months) zebrafish [26]. The calorie-restricted diet decreased body weight for both age groups and shortened telomere length of young zebrafish in the spleen and brain.

### Inflammation and cardiovascular disease

Normal acute inflammation responses are localized, typically in response to tissue damage or infection [27]. Capillary dilation, heat, redness, cytokine and/or chemokine release, swelling, and leukocyte infiltration are all characteristics of this response. In contrast, chronic low-grade systemic inflammation is debilitating independently but can be caused by poor metabolic health [28].

The effects of diet in inflammatory response are one of debate within the nutrition community, and especially in the role of dietary lipids [29]. The lipid composition of the diet affects markers of inflammation in zebrafish. Fish provided an isocaloric diet of differing n3:n6 ratio (1:2, 1:5, or 1:8) had decreased expression of vitellogenin (males only), c-reactive protein, and serum amyloid A as the ratio of n3:n6 in the diet increased [30]. Zebrafish provided the highest n3:n6 ratio also had the highest body weight in females, but the lowest adiposity in both males and females. Diets formulated with a low n3:n6 ratio and high levels of w6 arachidonic acid (ARA) resulted in increased oxidative stress and lipid peroxidation [31]. The metabolomic analysis showed a lower whole body n3:n6 fatty acid ratio in relation to higher levels of eicosanoids. Though ARA-derived eicosanoids are considered proinflammatory, no markers of inflammatory response were reported in these fish.

Dietary-induced obesity in zebrafish has been shown to impact inflammatory pathways. Comparative transcriptomics of visceral white adipose in zebrafish and mammals (rats, mice, and humans) show that genes responsible for blood coagulation and platelet activation are dysregulated in obesity [12]. Regulators of these genes include *il-6*, *il-1β*, and *apoh*, which act as proinflammatory cytokines leading to chronic inflammation. These cytokines also play a role in the initiation and promotion of certain types of cancers. Forn-Cuní et al. [32] reported the impact of dietary-induced obesity on the liver transcriptome following an injection of an inflammatory stimulus (lipopolysaccharide). Comparisons between non-obese zebrafish receiving inflammatory stimuli and non-obese controls showed increased expression of *pamp*, *tlr5b*, proinflammatory cytokines *il-1β* and *il-8*, and chemokines *cxcl-c1c* and *cxcl-11 l*. Injection of inflammatory stimuli in obese zebrafish showed none of the changes compared to obese controls. These data suggest an inability in obese fish to support an appropriate inflammatory response when a stimulus for such a response is present. One of the genes displaying increased expression in obese zebrafish compared to non-obese controls was *tac4*. This gene is still categorized as unknown function in zebrafish, but a paralog of this gene has a role in chronic inflammation [33]. Karanth et al. studied the impact of isocaloric and isonitrogenous diets of either 4% or 12% lipid diets on atherosclerotic cardiovascular disease [34]. The 12% lipid diet resulted in higher body weight and body length in male, but not female zebrafish. The activity of the enzyme HMGCR was reduced in both males and females fed the 12% lipid diet. HMGCR is involved in LDL clearance and is a potent target for pharmacological inhibitory intervention to reduce cardiovascular-related mortality [35].

In aquaculture, fish meal is a common ingredient which serves as a protein source; however, attempts to replace this source with a more cost-effective, available, and purported eco-friendly protein source, such as soy, are being considered [36]. Inclusion of dietary soy has stimulated research into the growth and health effects of fish protein replacement by soy sources, especially inflammatory responses due to the immunogenic effects of soy components. Hedrera et al. created diets using different protein sources of either fish meal, fish and soybean meal, fish meal and soy protein isolate, or fish meal and soy saponins [37]. Diets containing soybean meal or saponins resulted in increased neutrophil infiltration into the intestines and increased expression of the inflammatory cytokine *il-8*. All diets containing soy components increased *il-1β* expression as well. Fuentes et al. also formulated diets of fish meal, fish meal with low and high content soy protein isolate, or fish meal with low and high content of soy saponins [38]. These diets showed that there are dose thresholds for inflammatory impacts of soy diet components. The diets with high inclusion of soy diet components resulted in increased granulocyte number in regions of the digestive tract and increased proinflammatory cytokines and peroxidases. Both studies use larval stage (5–10 dpf) zebrafish, but these effects of soy dietary components are shown to persist in later life. Early exposure to these dietary components modulates exposure in adulthood in a type of nutrient programming in which the zebrafish model can be used to investigate further [39]. Ulloa et al. investigated the impacts of plant (a mix of soy protein, wheat gluten, and corn gluten) and fish protein diets on zebrafish growth and expression genes related to muscle growth [40]. Muscle expression of *igf2a* was decreased and *myogenin* and *mrf4* increased in males provided the plant protein diets, while female expression was not changed. Differences in growth-related genes were also found among isolated zebrafish families in response to plant and fish protein diets.

Cholesterol content is high in western diets and contributes to increased circulating cholesterol and risk of atherosclerosis and coronary heart disease [41, 42]. In Yoon et al., zebrafish provided a high-cholesterol diet that showed inflammatory responses and increased *il-1β* expression, but only in reproductive adults [43]. Reproductive adults in a study by Progatzky *et al.* [44] fed a high-cholesterol diet did show an inflammatory response, but no significant change in *il-1β* expression. This raises questions as to the pathways required for an inflammatory response to be produced and if dietary cholesterol impacts inflammation directly or via a non-canonical, inflammatory-regulating pathway in zebrafish. We should point out that there were differences in the feeding regime used in these studies. Cholesterol can contribute along with other dietary compounds to an inflammatory response. Aspartame provided in the zebrafish diet resulted independently in inflammation in the liver and brain, determined by oil-red O and hematoxylin staining [45]. When provided a diet that contained high cholesterol and aspartame, a synergistic increase in inflammatory responses was observed in these tissues. A high-cholesterol diet altered the lipid profile of zebrafish 5 to 14 days post-fertilization with a 70-fold increase in oxidized cholesterol esters [46]. A homogenate prepared from these zebrafish increased murine macrophage cell surface area and resulted in increased phosphorylation of ERK1+2, AKT, and JNK. Stoletov et al. [47] studied high-cholesterol diets to understand inflammation and arterial lipid accumulation. A high-cholesterol diet resulted in increased circulating cholesterol, altered lipoprotein profile, and increased macrophage activity in transplanted murine myeloid cells. High cholesterol also increased PLA2 activity, which is associated with increased CVD risk in humans by an unknown mechanism [48]. A high-cholesterol diet increased circulating cholesterol, triglycerides, and glucose and raised cholesterol-ester transfer protein (CETP) activity compared to a control diet [49]. Inclusion of a high-cholesterol diet with dried acai, a potent anti-oxidant berry of the acai palm, decreased circulating cholesterol and glucose compared to the high-cholesterol diet alone and fully returned CETP activity to the lower activity level of the control diet. Higher CETP activity is related to cardiovascular disease in some populations [50]. Pharmacological treatments to inhibit CETP activity in clinical trials have had many negative results or are ongoing [51]. Two cholesterol maintenance drugs commonly prescribed, ezetimibe and simvastatin, ameliorate the high circulating levels of cholesterol that occurs in zebrafish when they are fed a high-cholesterol diet [52], further supporting the use of zebrafish in translational research.

### Bioactive dietary compounds

Zebrafish have recently been used to explore dietary bioactive compounds that impact disease onset and progression. Anserine and creatine, reported as anti-obesity therapeutics from mice studies [53, 54], were proffered to overfed zebrafish DIO models to explore hepatic gene expression leading to lipid metabolism alterations in obese phenotypes with related comorbidities [55]. These bioactive food compounds ameliorated the impact of an obesogenic diet on symptoms of metabolic disorders such as high blood glucose, dyslipidemia, and hepatic steatosis and normalized expression changes of genes related to lipid metabolism. When zebrafish were provided an obesogenic diet of increased calories and containing green tea extract (GTE), protective effects were observed [56]. In both males and females, visceral and total body fat increased when fish were provided an obesogenic diet, but the inclusion of GTE reduced visceral and total body fat increases. Males and females both increased total body weight on the obesogenic diet, but only females decreased in total body weight compared to DIO fish when GTE was included in the diet. The highest dietary inclusion of GTE in females increased liver expression of the lipid catabolism gene *acox1*, *acadm*, and *ppara* and decreased *socs3* adipose expression, which affects leptin levels. Another study on the effects of GTE showed females provided a high-fat diet supplemented with GTE had increased citrate synthase and 3-hydroxyacyl-coenzyme A dehydrogenase activity compared to females fed a high-fat diet without GTE supplementation [57]. Zebrafish provided an obesogenic diet with the inclusion of Campari tomatoes (high in lycopene and beta-carotene) showed reduced weight gain and reduced triglycerides [58]. Expression of a number of genes relating to lipid metabolism, carbohydrate transport, and cell cycling, among others, was altered in the liver. Peels of the Japanese citrus fruit Yuzu (*Citrus junos*), which contains several bioactive compounds, also impacted DIO fish by reducing triglycerides and hepatic steatosis [59]. In the liver *pparab* and its targets *acox1* and *acadm* were upregulated, and in adipose *pparg*, *acox1*, and *adipoqb*, which regulate adipose differentiation, were also upregulated in fish provided an obesogenic diet with Yuzu fruit peel supplementation compared to fish given a non-supplemented obesogenic diet. Caffeine has been shown to ameliorate the hepatic effects of overfeeding and decreases weight, circulating triglycerides, and steatosis [60]. Caffeine also regulated expression of lipogenesis genes *aco*, *srebp1*, *acc1*, *cd36*, and *ucp2*; endoplasmic reticulum stress genes *perk*, *ire1*, *atf6*, and *bip*; inflammatory cytokines genes *il-1β* and *tnfa*; and autophagy genes *atg12* and *beclin-1*.

### Carbohydrate metabolism and β-cell function

Fang et al. evaluated impacts of a high-carbohydrate (60% maltodextrin), low-protein dietary challenge in early life time-periods (larval stages between 3 and 10 days post hatch), and nutritional programming effects at 16 weeks with a high-carbohydrate low-protein dietary challenge (35% maltodextrin) [61]. In early life dietary challenges, expression of carbohydrate metabolism-related genes including *gk*, *pk*, *g6pase*, *amy*, *pepck*, and *sglt-1* was differentially regulated in an age- and dietary-dependent manner. The dietary challenges at 16 weeks suggest metabolic programming by the initial high carbohydrate exposure. Both gene expression of all prior evaluated genes (except for *g6pase*) and activities of their respective enzymes were dependent upon initial exposure to differing dietary challenge. Rocha et al. [62] examined the consequences of early high carbohydrate exposure modulates later response by injecting zebrafish embryo yolks at 1-dpf with glucose or a saline vehicle and at 24-dpf they were provided a high-carbohydrate diet. Zebrafish that had received the 1-dpf glucose injection had decreased *pkl* and increased *hk1* expression in viscera and increased *6pfk* expression in muscle, all of which are related to alterations in gluconeogenesis. Seiliez et al. also looked at diets with differing protein and carbohydrate ratio by refeeding with either a high-protein, low-carbohydrate diet (HPLC) or a low-protein, high-carbohydrate diet (LPHC) following a 72-h starvation period [63]. Refeeding with LPHC increased hepatic *gk* and *pk* expression compared to HPLC refeeding and decreased expression of muscle *acca*, which functions in lipid metabolism.

In zebrafish, carbohydrate metabolism can be affected by the route of exposure. Different methods of studying the impact of carbohydrate metabolism changes exist. Exposure can be accomplished by diet, glucose injection, or a transdermal exposure to a high glucose environment, all of which can result in hyperglycemia [64–66]. Though non-dietary studies are less directly relevant to nutrigenomics, changes in gene regulation are important and some can be discussed due to the relevant regulatory changes and nutritional programming in metabolic disorders and due to the lack of a current dietary model for insulin resistance in zebrafish. Blood glucose has been shown to increase with exposure to a high-glucose environment [67]. Following removal from a high-glucose environment, blood glucose was maintained high during a 7-day wash-out period in clean system water. Treatment with the drugs glimepiride and metformin, popular clinical treatments for type 2 diabetes, reduced blood glucose back to normal levels. Along with increasing blood glucose, transdermal glucose exposure increased *insra-1*, *insrb-1*, and *insrb-2* expression in the skeletal muscle. Another study showed the drug glipizide also proved effective at reducing elevated blood glucose caused by transdermal exposure [68]. Larval exposure to transdermal glucose reduced the expression of *pepck* and increased expression of *insa.* Injection of the diabetogenic drug streptozocin reduces insulin secretion and blood glucose levels, mimicking type-1 diabetes with zebrafish recovering from these effects within 14 days of injections ending [69]. Though recovery from environmental glucose exposure occurs, differences in genome-wide CpG island methylation changes and expressions persist. Caudal fin amputation following environmental glucose exposure shows that regeneration of the tissue is compromised, and new tissue has similar changes in expression and methylation patterning considered to be metabolic memory.

The function of β-cells of the pancreas is very important for carbohydrate metabolism and these cells respond to dietary challenges in zebrafish. Maddison et al. provided media with high amounts of either glucose or lipid (from locally sourced egg yolks) to larval stage fish either intermittently or persistently [70]. Either diet provided persistently increased the number of β-cells by initiating differentiation of precursor cells. The high glucose and high lipid exposure acted via different mechanisms to initiate differentiation, with the mTOR pathway being required for the response to the glucose-rich diet and the IGF-1 signaling being required for the response to the lipid-rich diet. Ninov et al. [71]. found similar results for excess nutrient availability for larval zebrafish by providing diets containing higher carbohydrate and lipid content which resulted in increased β-cell proliferation and in differentiation of progenitor cells in the pancreatic duct. The mechanism for these nutrient-driven changes was shown to be mTOR dependent. This persistent overnutrition effect on β-cell number is also supported by Michel et al. who utilized a high lipid exposure protocol [72].

Another study looking at steatosis formation used 5–7 dpf larval zebrafish exposed to either 4% glucose or 4% fructose treatments [73]. The fructose treatment resulted in increased steatosis and micrograph examination showed dilated endoplasmic reticulum, a sign of ER stress, and a “less distinct” mitochondrial membrane. The fructose treatment also increased expression genes related to lipogenesis (*cidec*, *lipin1*, *lipin2*, and *srebpf1*), inflammation (*tnfa*, *irf2a*, and *nfkb*), oxidative stress (*gpx* and *trxr2*), and ER stress (*ddit3*). Treatment with rapamycin to inhibit mTOR ameliorated the hepatic steatosis and all the genetic expression changes except for *irf2a* and *nfkb*.

### Tumorigenesis and dietary carcinogen exposure

Methyl mercury is an environmental contaminant that can enter the body by multiple pathways including dietary contamination. Exposure to methyl mercury-tainted feed for 25 days resulted in differential gene expression patterns in zebrafish skeletal muscle [74]. Expression was altered in genes affecting general cell metabolism, lipid metabolism, cell cycle regulation, and ribosomal components involved in protein synthesis. The multiple ribosomal genes evaluated in this study had expression changes correlated to colorectal carcinoma, adenocarcinomas, and DNA integrity, though not in the skeletal muscle [75–78].

Polycyclic aromatic hydrocarbons (PAHs) are another class of environmental contaminants that can enter the body via dietary exposure. A diet contaminated by PAHs decreased survival and increased formation of global neoplasms with bile duct epithelial being the most susceptible [79]. Expression of the gene *cyp1a*, which is related to both circadian rhythm and detoxification, increased following ingestion of the PAH contaminated diets [80]. Expression of *ahr2*, which codes for a receptor that binds aromatic compounds, was unaffected by the PAH-contaminated diets except at the highest concentration where repression was observed [81]. Zebrafish consuming feed contaminated with TCDD (dioxin) resulted in dose- and time-dependent bioaccumulation and formation of lesions in multiple organs [82]. Microarray produced expression changes related to several genetic ontologies and pathways including cardiac fibrosis, lipid transport, metabolic processes, DNA replication, as well as cardiac, renal, and liver necrosis among others. A diet containing the 2,4-dimethoxybenzaldehyde (DMBA) resulted in a dose-dependent increase in weight, neoplasm formation, and mortality [83]. A diet contaminated with the carcinogen methylnitronitrosoguanidine showed no impact on zebrafish weight, survival, or neoplasm formation [84]. This was different from the profound effects of transdermal exposure or injection that formed neoplasms at a low dosage.

### Vitamin and mineral deficiency

Retinoic acid (RA), one of the forms of vitamin A, was supplemented in diets for adult zebrafish [85]. Adult female zebrafish were initially fed a control diet and then transferred to either control, RA-supplemented diet, control diet and DEAB (which inhibits de novo RA synthesis), or control diet and DEAB with the RA supplement. All females that were treated with DEAB had reduced egg production 5 days after the diets were changed suggesting low RA levels inhibit egg production. The RA-supplemented diet maintained egg production similar to the control diet until 9 days after the diet was changed, and suggested an over supplementation of RA inhibits egg production. In male zebrafish testes, but not female ovaries, the RA supplemented diet decreased *raldh2* expression, an enzyme that synthesizes retinal acid from retinol, and increased *cyp26a*, an enzyme that converts retinoic acid to polar metabolites for excretion [86, 87].

Zebrafish are one of the few species that lack gluconolactone oxidase that converts gluconolactone to ascorbic acid, though zebrafish need vitamin C for many processes [88]. Kirkwood et al. formulated diets deficient in vitamin C, vitamin E (tocopherol), and both vitamin E and vitamin C [89]. Both vitamins have antioxidant activity and can provide a sparring effect on each other. The vitamin C-deficient diet resulted in increased oxidative stress and increased AMPD enzyme activity, necessary for purine nucleotide synthesis and cellular energy [90]. Levels of several metabolites also changed in response to the vitamin C-deficient diet including metabolites related to amino acids and amino acid derivatives, carnitine metabolism, glutathione synthesis, glycerophospholipid synthesis, and purine metabolism. Aside from the role of vitamin E in vitamin C sparing, the response of parental vitamin E in offspring has been studied in zebrafish. Miller et al. provided a commercial lab diet, a diet with vitamin E supplementation, or a diet deficient in vitamin E to reproductive age adults [91]. Offspring from adults on the vitamin E-deficient diet had increased malformations at 2 and 3 days post fertilization and lower levels of tissue vitamin E. Microarray showed 2656 genes differentially expressed between the offspring of the vitamin E-deficient and vitamin E-supplemented diets. Several biological processes were altered including embryonic development, cell development, tissue development, cell growth, and cell cycle.

Zinc supplementation in the system water and the diet significantly increased body zinc and caused differential expression of 525 genes in zebrafish gills [92]. Genes related to transcription factors and steroid hormone receptors were enriched, impacting multiple pathways related to growth. Temporal transcriptome analysis showed that gene expression changes occurred immediately following transfer to water containing elevated levels of zinc, and the response to dietary zinc supplementation was maximal by day 7. By day 14, most of the genes impacted by zinc supplementation returned to basal expression levels. Beaver et al. provided zinc-deficient diets to zebrafish and looked at the impact on their offspring [93]. Embryos produced by those receiving the zinc-deficient diets had increased embryonic mortality and malformations of the snout and eyes. They also showed altered expression of genes related to metal homeostasis (*znt8*, *znt9*, and *mtf1*), diabetes and pancreatic development (*insa*, *pax4*, and *pax5*), and DNA methylation (*dnmt4*, and *dnmt6*). All of these show a temporally dependent expression change. These alterations of expression, especially those that impact DNA methylation and organ development, can have lasting impacts and shows the importance of zebrafish as models for maternal and offspring dietary studies. Diets supplemented with sodium selenite pentahydrate increased body selenium levels in male and female zebrafish after 7 days and altered the selenoproteome of the brain [94]. Selenoproteome alterations were strongly dependent on time and additional qRT-PCR shows differences between sexes.

### Circadian rhythms

Recently, zebrafish have been used alongside more traditional animal models to understand circadian rhythms. Circadian rhythms are driven by primarily endogenous molecular events, occurring in 24-h cycles, which can be impacted by environmental factors such as energy intake and light exposure. Zebrafish held under a 24-h constant light exposure, as compared to the typical 14-h:10-h light and dark cycle, have an altered expression of genes that are important in maintaining circadian cycles such as *clock*, *per1*, *per2*, and *cry1a* [95]. *Clock*, *per1*, and *cry1a* are also dysregulated by providing the animals with a high-fat diet. High-fat diet also altered the expression of several *ppar* isoforms and *lpl*, while a continuous light cycle only altered *pparbd* expression. These results demonstrate the value of the zebrafish model to understand intersecting molecular pathways between circadian rhythms and metabolic disorders.

### Bone Formation

High-fat diets in zebrafish affect bone formation [96]. Calcein staining showed decreased bone mineralization around the border of the scales. The activity of alkaline phosphatase is decreased and the activity of tartrate-resistant acid phosphatase is increased. Expression of *thfrsf11* and *thfrsf11/thfrsf11b* ratio are both increased, which suggests impacts on bone reabsorption. Along with these changes in bone formation, zebrafish on the high-fat diet treatment exhibited decreased adiponectin and increased leptin, weight, BMI, and advanced glycation end products.

## Conclusions

The case for the importance of the zebrafish model in nutrigenomic studies is substantial. Zebrafish models have been developed for almost any human disease in which nutrition is a confounding factor. The results in these studies are easily translatable to other animal models or human intervention trials for a wide array of public health concerns.

As the zebrafish model continues to be utilized in nutrigenomic studies, certain considerations will maximize effectiveness and create new avenues for investigation. Defined reference diets, similar to those used in rodents, are being developed to enhance experimental rigor and reproducibility. Currently, many labs rely on commercially available diets that can have unknown composition and differing sourced ingredients. These ingredients can contain undetected or unreported bioactive dietary components. The zebrafish community will benefit from an increased understanding of the nutritional needs of zebrafish [97], and additional research and education should be forthcoming.

Manipulation of diet content is an important tool to investigate diet-related physiology and molecular changes. Age-related responses to dietary content should also be considered in current and future studies. Studies of reproduction performed at various age structures and using various feed management strategies can yield different results. Alsop et al. bred females every few days to observe egg production following vitamin A supplementation [85]. Reproductive output was lower in zebrafish fed smaller rations compared to those fed large rations [98]. Ages at which diets were provided varied substantially among these studies. Temporal transcriptome studies reported by Zheng et al. demonstrated the importance of timing on genomic outcomes [92]. Thus, understanding the complexity of nutrigenomic outcomes in response to nutrient and non-nutrient-related manipulations will allow us to further clarify the role of nutrients in metabolic processes related to normal and disease states of zebrafish health.

Lastly, the value of zebrafish nutrigenetics studies has been enhanced by the high throughput nature of the model. Zebrafish have been used for drug discovery and forward genetic screens, and for novel gene functions and impacts of genetic variation [99, 100]. Similar screening of dietary components that function differentially in specific genotypes is not only possible but scientifically necessary and highly valuable. Parks et al. investigated the effects of diet on over 100 inbred strains of mice, yielding important information on single nucleotide differences in diet response. Similar study designs can be performed in the zebrafish model with less logistic effort to discover novel diet-responsive alleles [101]. Recently, single nucleotide polymorphisms of several growth factor genes were identified following the consumption of plant protein diets and were implicated as novel targets for future investigation [102]. Large-scale transposon insertion and targeted gene-editing techniques that have recently been used with zebrafish also provides means of novel gene discovery and of how gene variants can alter responses to diet.

The value of the zebrafish as a nutrigenomic model is just now being discovered. We hope that more studies will focus on the use of the zebrafish model for gene-diet interactions, and we believe that this information will be translatable to human health.

## Data Availability

Not applicable
